# Untargeted Metabolomics Showed Accumulation of One-Carbon Metabolites to Facilitate DNA Methylation during Extracellular Matrix Detachment of Cancer Cells

**DOI:** 10.3390/metabo12030267

**Published:** 2022-03-21

**Authors:** Suza Mohammad Nur, Mohammed Razeeth Shait Mohammed, Mazin A. Zamzami, Hani Choudhry, Aamir Ahmad, Bushra Ateeq, Irfan A. Rather, Mohammad Imran Khan

**Affiliations:** 1 Biochemistry Department, Faculty of Science, King Abdulaziz University, Jeddah 21589, Saudi Arabia; snur0001@stu.kau.edu.sa (S.M.N.); razeeth.new@gmail.com (M.R.S.M.); mzamzami@kau.edu.sa (M.A.Z.); hchoudhry@kau.edu.sa (H.C.); 2 Centre of Artificial Intelligence for Precision Medicines, King Abdulaziz University, Jeddah 21589, Saudi Arabia; 3 Translational Research Institute, Academic Health System, Hamad Medical Corporation, Doha 3050, Qatar; aahmad9@hamad.qa; 4 Molecular Oncology Laboratory, Department of Biological Sciences and Bioengineering, Indian Institute of Technology Kanpur, Kanpur 208016, Uttar Pradesh, India; busatiik_11@yahoo.com; 5 Department of Biological Sciences, Faculty of Science, King Abdulaziz University, Jeddah 21589, Saudi Arabia

**Keywords:** one-carbon metabolism, ECM detachment, anoikis, DNA methylation, NKG2DLs

## Abstract

Tumor cells detached from the extracellular matrix (ECM) undergo anoikis resistance and metabolic reprogramming to facilitate cancer cell survival and promote metastasis. During ECM detachment, cancer cells utilize genomic methylation to regulate transcriptional events. One-carbon (1C) metabolism is a well-known contributor of SAM, a global substrate for methylation reactions, especially DNA methylation. DNA methylation-mediated repression of NK cell ligands MICA and MICB during ECM detachment has been overlooked. In the current work, we quantitated the impact of ECM detachment on one-carbon metabolites, expression of 1C regulatory pathway genes, and total methylation levels. Our results showed that ECM detachment promotes the accumulation of one-carbon metabolites and induces regulatory pathway genes and total DNA methylation. Furthermore, we measured the expression of well-known targets of DNA methylation in NK cell ligands in cancer cells, namely, MICA/B, during ECM detachment and observed low expression compared to ECM-attached cancer cells. Finally, we treated the ECM-detached cancer cells with vitamin C (a global methylation inhibitor) and observed a reduction in the promoter methylation of NK cell ligands, resulting in MICA/B re-expression. Treatment with vitamin C was also found to reduce global DNA methylation levels in ECM-detached cancer cells.

## 1. Introduction

Extracellular matrix (ECM) attachment is required for cell survival [1], whereas ECM detachment induces the programmed cell death called anoikis. Anoikis is considered a cancer hallmark, and during oncogenic progression, cancer cells deregulate anoikis and overcome it; hence, the cells become anoikis-resistant [2,3]. Moreover, ECM detachment intimately assists in cancer metastasis and ensures metabolic reprogramming with metastasis progression [4,5,6]. For example, a research study revealed that ECM detachment could induce the reductive carboxylation metabolism that promotes glycolysis [7]. Furthermore, loss of cell matrix attachment can induce hypoxia [7], which, in turn, is able to adapt tumor metabolism [8,9], though inductive correlations between hypoxia, ECM remodelling, and metastasis exist [10,11]. In addition, a well-founded study also revealed that the ratio of S-adenosyl methionine (SAM) to S-adenosyl homocysteine (SAH) (SAM/SAH ratio) is a tightly regulated epigenetic modification, as is DNA methylation [12]. The distribution of DNA methylation occurs predominantly (60–80%) in CpG dense regions, while 10% occurs in CpG islands (CGIs) [13]. The primary mechanism of DNA methylation underlies the transfer of SAM metabolites’ methyl groups through covalent modification, with one carbon atom attaching to the fifth position at cytosine to form 5-methyl cytosine (5mC) [14,15]. In addition, this covalent modification is mainly driven by a group of DNA methyltransferase enzymes known as DNMT1, DNMT3a, and DNMT3b; DNMT1 maintains the methylation reaction during the replication phase and the other two DNMTs perform de novo methylation [16,17]. Recently, our research team has identified that ECM detachment results in a metabolic rearrangement that could promote levels of metabolites associated with methylation, including S-adenosyl methionine (SAM) and S-adenosyl homocysteine (SAH) [18]. However, the global landscape of pathways related to methylation is still incompletely mapped, with certain pathways missing. Given this situation, our study aims to explore the one-carbon metabolism that supports the methylation of DNA/RNA and proteins.

Natural killer (NK) cells are innate immune cells and, in physiologically damaged conditions, such as tumor cell growth, they act as robust cytotoxic functioning cells. Although healthy cells express MHC-I, an inhibitory ligand for NK cells, cancer cell surfaces lose this molecule [19,20]. Moreover, NK cell-expressed activating receptor (natural killer group 2D) NKG2D captures MHC-I-type ligands on cell surfaces [21], rendering immune surveillance against tumors [22]. One of the most studied and genomically prevalent human ligands is MHC class I polypeptide-related protein A, along with B-MICA [23] and MICB [24]. However, NKG2DL expression has been reported in several pooled scientific data sets as being under tight epigenetic regulation [25,26,27]. A study also revealed that expression may be absent due to DNA methylation-mediated transcriptional silencing [28]. However, epi-transcriptomic silencing could be overcome and expression activated upon treatment with DNMTis, such as DAC and 5azaC [29]. In addition, a recent review revealed that vitamin C could reprogram the epigenome [30,31].

However, the study also showed that MICA expression is reduced when a cell loses its adherence matrix [32]. It has been overlooked whether the upregulated DNA methylation in ECM-detached cancer cells could repress MICA/B expression. ECM detachment can cause hypoxia [7], which mediates the DNA hypermethylation [33]. Therefore, this study has also been designed to investigate DNA methylation status and MICA/B protein expression in both attached and detached conditions with or without 5-aza-dc and vitamin C treatment.

## 2. Results

### 2.1. Loss of ECM Attachment Increased Levels of One-Carbon (1C) Metabolites

**Figure 1:**
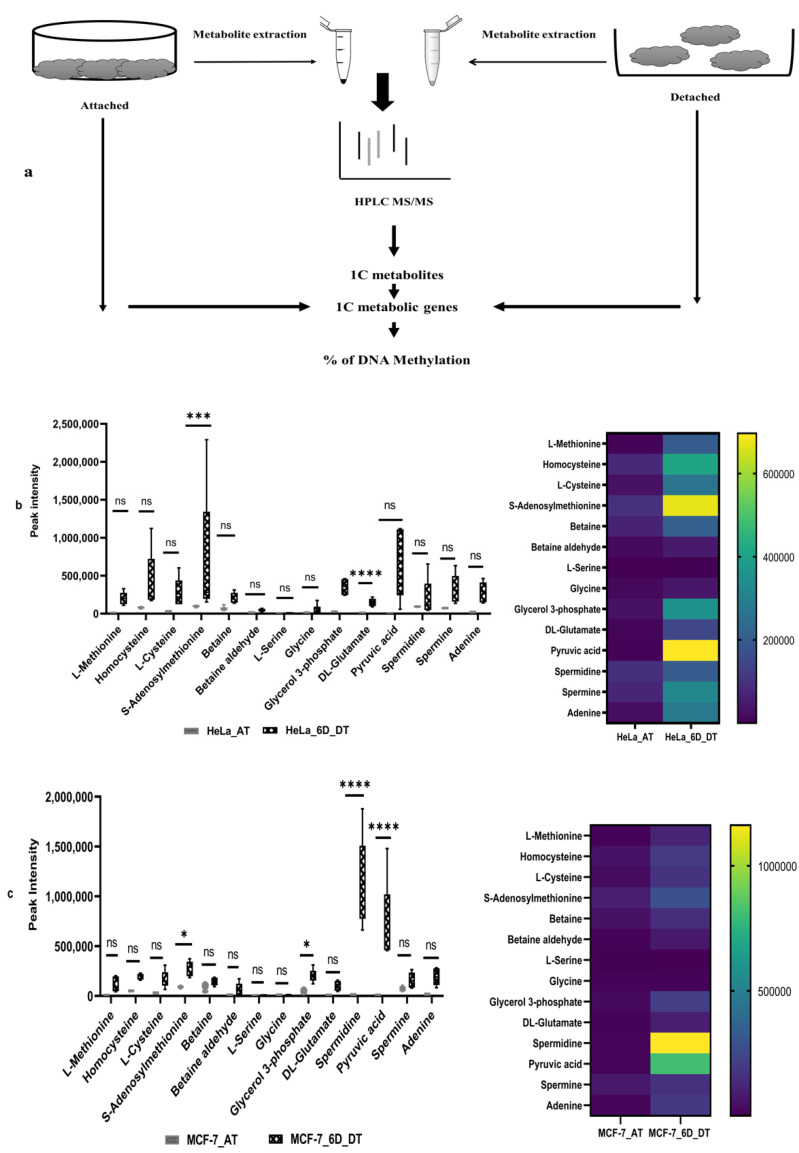
Increasing the level of one-carbon metabolites in the ECM-detached condition. (**a**) Overall experimental design. (**b**) The one-carbon metabolites detected in the attached (AT) and 6 days (6D) ECM-detached HeLa cell line. (**c**) The one-carbon metabolites detected in the attached (AT) and 6 days (6D) ECM-detached MCF-7 cell line. The data are presented as mean ± SEM; ns, not significant; * *p* < 0.05, *** *p* < 0.001 and **** *p* < 0.0001.

First, both HeLa and MCF-7 cell lines were grown in poly-HEMA gel-coated 6-well plates for six days to induce the ECM detachment model in cell lines. After that, both HeLa and MCF-7 cell lines, attached and 6 days ECM-detached cell aliquots, were taken for whole experiments (Figure 1a). The extracted metabolites were detected by HPLC coupling with LTQ LC–MS-based high throughput technology used to generate patterns of fragments and identify untargeted metabolites. The metabolite spectrum has been obtained through the data-dependent acquisition (DDA) method, followed by the processing of RAW files. A total of 833 metabolites was detected in both cell lines, and screening was performed to make shorts out of metabolites related to the one-carbon metabolic pathway. The peak intensity of these metabolites was plotted for a box plot and heat map (Figure 1b,c). Our results show that, after 6 days of ECM detachment in the HeLa cell line, higher levels of one-carbon metabolites, such as SAM, methionine, cysteine, homocysteine, etc., except L-serine, were detected. In addition, higher levels of glycerol-3-phosphate, DL-glutamate, pyruvic acid, adenine, spermine, etc., were also detected in the HeLa cell line (Figure 1b).

In contrast, the peak intensity results for the ECM-detached MCF-7 cell lines showed that they also accumulated higher levels of core one-carbon metabolites, such as SAM, methionine, cysteine, homocysteine, spermidine, pyruvic acid, adenine, etc. Compared with the ECM-detached HeLa cell line, the ECM-detached MCF-7 cell line had accumulated a higher level of core one-carbon metabolites to a lesser extent (Figure 1b,c). However, spermidine was highly detected in ECM-detached MCF-7 cell lines.

### 2.2. Loss of ECM Attachment Increased Expression of One-Carbon Pathway-Related Metabolic Genes and Global DNA Methylation

**Figure 2:**
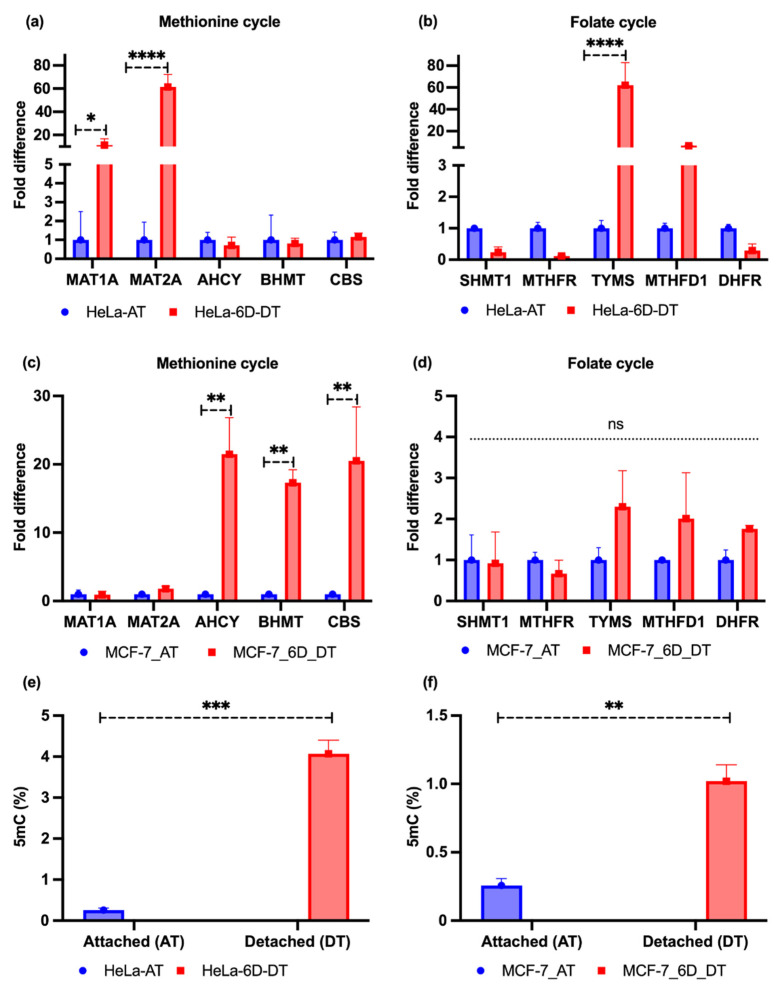
One-carbon metabolic genes and 5mC levels (%) in ECM-attached and -detached conditions. (**a**) The mRNA expression levels of one-carbon metabolic genes in the attached (AT) methionine cycles and 6 days detached (6D_DT) conditions in the HeLa cell lines. (**b**) The mRNA expression levels of one-carbon metabolic genes in folate cycles in attached (AT) and 6 days detached (6D_DT) conditions in the HeLa cell lines. (**c**) The mRNA expression levels of one-carbon metabolic genes in methionine cycles in attached (AT) and 6 days detached (6D_DT) conditions in the MCF-7 cell lines. (**d**) The mRNA expression levels of one-carbon metabolic genes in folate cycles in attached (AT) and 6 days detached (6D_DT) conditions in the MCF-7 cell lines. SHMT1, serine hydroxymethyltransferase 1; MTHFR, methylenetetrahydrofolate reductase. (**e**,**f**). HeLa and MCF-7 cell lines showed increased 5mC levels in the 6 days (6D_DT) ECM-detached condition compared to the attached (AT) condition. The data are presented as mean ± SEM; * *p* < 0.05, ** *p* < 0.01, *** *p<* 0.001, and **** *p* < 0.0001.

We cultured the attached HeLa and MCF-7 cell lines in T75 cm^2^ flasks. Simultaneously, they were grown in poly-HEMA-coated 24-well plates for 6 days. After extracting RNA, cDNA conversion was performed for conducting qRT-PCR. Our experimental results showed that the methionine cycle-driving genes, such as methionine adenosyltransferase-1A/2A (*MAT1A/2A*), were significantly upregulated in the HeLa cell line. In addition, two genes (~33%) involved in the folate cycle were induced; the thymidylate synthetase (*TYMS*) gene was highly induced, while methylenetetrahydrofolate dehydrogenase 1 (*MTHFD1*) was slightly induced. The cystathionine β-synthase (*CBS*) gene was marginally induced. The rest of the genes were downregulated (Figure 2a,b). However, the MCF-7 cell line results showed that the genes involved in the methionine cycle, such as betaine homocysteine, S-methyltransferase (*BHMT*), and S-adenosyl homocysteine hydrolase (*AHCY*), were upregulated, with marginal induction of *MAT1A/2A* genes. Only the *CBS* gene was found to be upregulated among the folate cycles genes, with marginal induction of dihydrofolate reductase (*DHFR*), *TYMS*, and *MTHFD1* (Figure 2c,d). Moreover, it is well-established that DNA methylation is marked by couples of DNA methyltransferase enzymes that obtain a methyl group from the donor metabolite S-adenosyl methionine (SAM) and covalently add to the 5′ position a cytosine base within the CpG region to form 5′-methylcytosine (5mC) [34]. Therefore, we performed a 5mC level test, in both attached and detached conditions, which revealed that, upon detachment from the ECM, MCF-7 and HeLa cell lines showed higher 5mC levels than attached cell lines (Figure 2e,f). Nevertheless, the levels in HeLa cell lines were about four times higher than those in the MCF-7 cell lines.

### 2.3. Loss of ECM Attachment Represses Expression of NKG2DLs by Inducing Promoter Methylation

**Figure 3:**
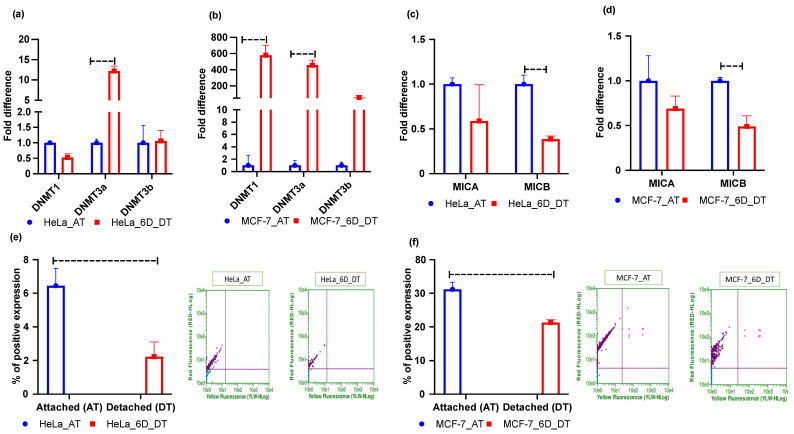
ECM detachment represses NKG2DLs–MICA/B expression. (**a**) Fold changes in mRNA expression of *DNMT*s in attached (AT) and 6 days ECM-detached (6D_DT) HeLa cell lines. (**b**) Fold changes in mRNA expression of *DNMT*s in attached (AT) and 6 days ECM-detached (6D_DT) MCF-7 cell lines. (**c**) Fold changes in mRNA expression of *MICA/B* in attached (AT) and 6 days ECM-detached (6D_DT) conditions in HeLa cell lines. (**d**) Fold changes in mRNA expression of *MICA/B* in attached (AT) and 6 days ECM-detached (6D_DT) conditions in MCF-7 cell lines. (**e**,**f**). MICA/B surface expression is repressed in the 6 days ECM-detached (6D_DT) condition compared to the attached (AT) condition in HeLa and MCF-7 cell lines. The data are presented as mean ± SEM; ns, not significant.

As our results showed increased levels of 5mC, global DNA methylation may also increase. Therefore, we checked the mRNA expression levels of *DNMT* genes. The results for HeLa and MCF-7 cell lines showed that ECM detachment induced mRNA expression of *DNMT*s in both cell lines (Figure 3a,b). However, MCF-7 cell lines had more *DNMT* mRNA induction than HeLa cell line (Figure 3b). Moreover, the innate immune system response by NK cells to counter cancer cell tumors necessitates interaction between NKG2D receptors and MICA/B ligands [25,35,36]. Hence, we performed qRT-PCR to assess *MICA/B* ligand mRNA expression in the 6 days ECM-detached MCF-7 and HeLa tumor cell lines. Our experimental results showed that *MICA/B* mRNA expression in MCF-7 and HeLa cell lines was repressed in ECM-detached conditions (Figure 3c,d). However, we also explored surface protein (MICA/B) expression through flow cytometry experiments and this showed that ECM detachment downregulated surface protein expression in both cell lines (Figure 3e,f).

### 2.4. Vitamin C—A Global Methylation Inhibitor Reduces DNA Methylation and Induces MICA/B Expression during ECM Detachment

**Figure 4:**
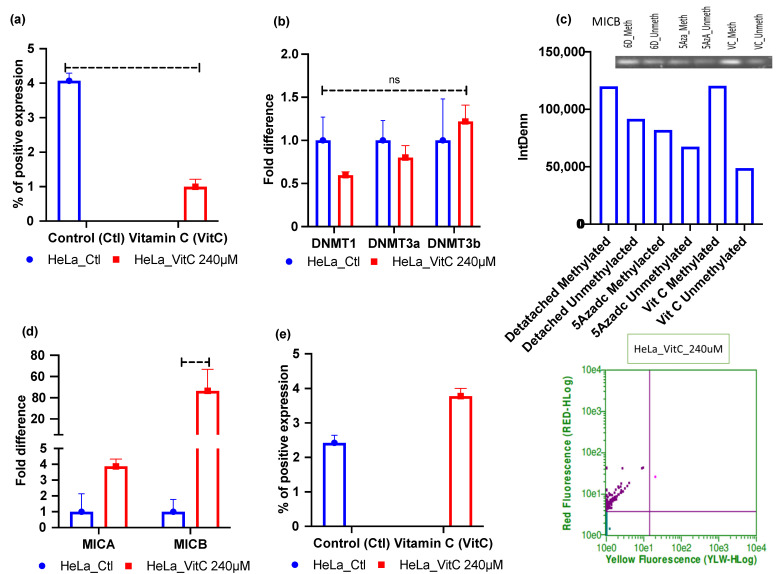
Vitamin C treatment mediated reductions in global DNA methylation, DNA methyltransferase (*DNMT1/3a*), and induction of *MICA/B* expression in ECM-detached HeLa cell lines. (**a**) Vitamin C treatment reduced 5mC levels. (**b**) Vitamin C treatment repressed DNMT1/3a mRNA expression. (**c**) HeLa cell line showing that 5-aza-dc and vitamin C did not reduce the methylation signal in gel electrophoresis compared to the untreated 6 Days ECM-detached condition. (**d**) Vitamin C treatment increased the fold changes in mRNA expression of *MICA/B* in the 6 days ECM-detached condition. (**e**) Vitamin C treatment increased surface MICA/B expression. 6D_Meth, 6 days detached, methylated; 6D_Unmrth, 6 days detached, unmethylated; 5Azadc_Meth, 5-aza-dc methyl-treated; 5Azadc_Unmeth, 5-aza-dc unmethylated; VC_meth, vitamin C methyl-treated; VC_Unmeth, vitamin C unmethylated. The data are presented as mean ± SEM; ns, not significant.

Vitamin C was found to be a potent epigenetic re-programmer in cancer and stem cells [37,38]; hence, we first assessed the 5mC levels of vitamin C-treated 6 days ECM-detached cells. Our results show that vitamin C treatment reduced the global methylation level in the HeLa cell line (Figure 4a). Furthermore, we also checked *DNMTs* mRNA expression by qRT-PCR for vitamin C-treated ECM-detached MCF-7 and HeLa cell lines. The results showed that treatment reduced *DNMT3a* and *DNMT1* expression in HeLa cell lines but that the treatment did not affect DNMT3b mRNA expression. The results were not statistically significant (Figure 4b). However, DNMTi treatment reduced mRNA expression of all the *DNMT*s (Figure S2). We also performed methylation-specific PCR experiments to explore and correlate methylation status. Our results showed that vitamin C did not reduce methylation-indicative signal intensity in HeLa compared to untreated 6 days ECM-detached cell lines (Figure 4c). After that, we also checked the status of the expression of NKG2DLs in vitamin C-treated ECM-detached cell lines and found that *MICA/B* mRNA and surface protein expression were induced in vitamin C-treated HeLa cell line (Figure 4d,e).

**Figure 5:**
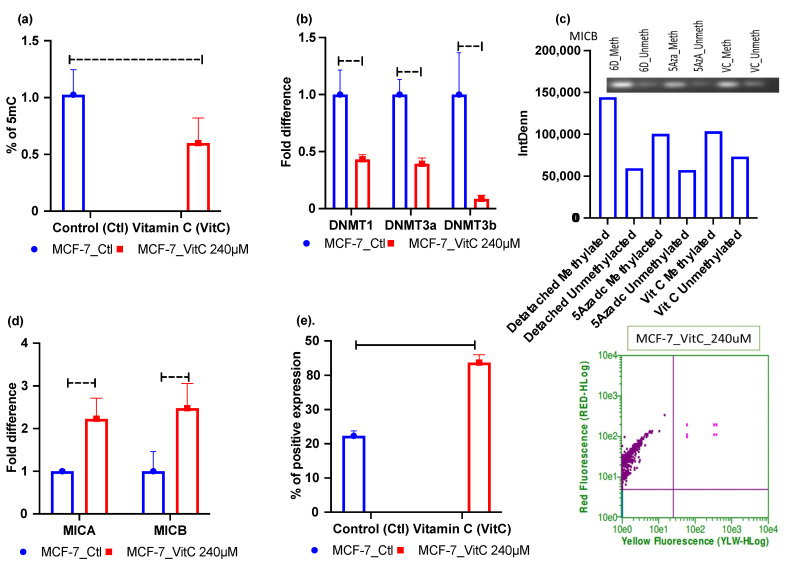
Vitamin C treatment-mediated reduction of global DNA methylation, DNA methyltransferase, and induction of *MICA/B* expression in ECM-detached MCF-7 cell lines. (**a**) Vitamin C treatment reduced 5mC levels. (**b**) Vitamin C treatment repressed *DNMTs* mRNA expression. (**c**) MCF-7 cell line showing 5-aza-dc and vitamin C reducing methylation signal in gel electrophoresis compared to untreated 6 Days ECM-detached condition. (**d**) Vitamin C treatment increased the fold change of *MICA/B* mRNA expression in the 6 days ECM-detached condition. (**e**) Vitamin C treatment increased surface *MICA/B* expression. 6D_Meth, 6 days detached methylated; 6D_Unmrth, 6 days detached unmethylated; 5Azadc_Meth, 5-aza-dc methyl-treated; 5Azadc_Unmeth, 5-aza-dc unmethylated; VC_meth, vitamin C methyl-treated; VC_Unmeth, vitamin C unmethylated. The data are presented as mean ± SEM; ns, not significant.

In contrast, in MCF-7 cell lines, vitamin C significantly repressed the global methylation level (Figure 5a). Not only that, vitamin C treatment also significantly hit and reduced mRNA expression of all *DNMT*s (Figure 5b). Moreover, the reduced methylation level in the *MICB* gene was confirmed by MSPCR experiments (Figure 5c). Additionally, vitamin C treatment-induced *MICA/B* mRNA and protein expression (Figure 5d,e). However, we could not detect methylation levels in *MICA* gene amplification on MSPCR. Since 5Aza-2-deoxycytidine (5-aza-dc) is known as a potent DNMT inhibitor [39], we chose 5-aza-dc as a positive control for our experimental treatments. (The data are presented in Supplementary Figures S1–S3.)

## 3. Discussion

Anoikis-resistant ECM-detached cancer cells undergo various metabolic adaptations and reprogramming to survive. For example, upon loss of ECM attachment, AMPK is induced to promote the uptake of glutamine, which lowers excessive oxidative stress. This metabolic reprogramming helps ECM-detached cancer cells to survive [40,41]. Moreover, tumor cells undergo epigenetic regulation, and the one-carbon metabolic pathway is linked to epigenetic modification, for instance, in DNA methylation reactions. This being so, the main aim of this study was to explore the relation between the one-carbon metabolic profiling and methylation-mediated transcriptomic regulation of the major innate immune ligands-MICA/B, in ECM-detached conditions. For this purpose, we performed untargeted metabolomics and sorted out the metabolites for the methionine and folate cycles of one-carbon metabolism. A recent study showed that loss of ECM attachment induced SAM and homocysteine levels in HCT116 and 22RV1 cancer cells. Although the SAM is a methyl group donor that activates methylation reactions in DNA, the study did not reveal the one-carbon metabolic pathway-mediated global methylation level in DNA [18]. Our study results demonstrated increased levels of SAM, methionine, and homocysteine in HeLa and MCF-7 cell lines. Most of the metabolites associated with 1C metabolism, such as glutamate, arginine, spermidine, and pyruvic acid, were also detected as higher metabolites.

Moreover, several previous studies have measured the expression levels of one-carbon metabolic genes among healthy cohorts, inflammatory macrophages, and liver cancer cells [42,43,44]. Another study elaborately revealed that one-carbon metabolites—methionine, SAM, m-THF, serine, etc.—and their associated enzymes are required by cancer cells for cell proliferation, DNA methylation reactions, and nucleotide synthesis [45]. Another study showed that controlling pyruvate by blocking its lactate-converting enzymes can slow cell proliferation in gastric cardia cancer clinical samples [46]. One-carbon metabolic pathway-mediated DNA methylation has already been targeted in cancer therapy, supported by several studies. For example, cell proliferation in HeLa cell lines decreased upon the downregulation of the folate-dependent DNA synthesis enzyme TYMS after 0.5 µM fluorouracil treatment with miRNA [47]. Similarly, the deregulation of folate cycle enzymes, such as the high expression of the *MTHFD1* gene, affects survival rates in acute leukemia patients [48]. Even with the knockdown of the *SHMT1* gene, there was evidence of apoptosis induction in lung cancer [49].

Moreover, therapeutic aspects have previously been revealed by several studies. For example, a study showed that 5-fluorouracil (5-FU—an inhibitor of TYMS) and methotrexate target the DHFR in ALL as a chemotherapeutic drug. Combined treatment with cyclophosphamide, 5-FU, and methotrexate showed effectiveness in node-negative breast cancer patients [50]. Another study revealed that 1C metabolism mediated DNA synthesis and DNA methylation can be used for cancer biomarkers, as well as one-carbon metabolic inhibitors can be useful in cancer therapy [51]. We examined these one-carbon metabolic genes in ECM-detached conditions. Our results indicated that expression levels of the genes responsible for SAM, m-THF, and dTUMP synthesis in ECM-detached HeLa cell lines were significantly high. However, methionine and homocysteine synthesis were slightly downregulated. The overall folate cycle was downregulated, except for trihydro-folate (THF) synthesis.

Similarly, the ECM-detached MCF-7 cell line also had high methionine, SAM, DHFR, and cystathionine synthesizing gene expression levels. The cell line also showed the slight induction of dTUMP, m-THF, and DHFR synthesizing gene expression. Moreover, both MCF-7 and HeLa cell lines showed that loss of ECM attachment induced 5mC levels, which indicated that DNMT activity could be induced [52]. A previous study also found increased 5mC levels in suspension carcinoma cells [53]. Hence, we aimed to check this effect of higher DNA methylation on the major innate immune cell ligands, especially the NK cell ligands MICA/B in the ECM-detached condition. We observed that ECM detachment induced *DNMT* mRNA expression and repressed *MICA/B* mRNA expression. Even the expression of surface proteins was downregulated after ECM detachment.

However, currently, natural compounds-based oncological therapy is not so widely available for cancer patients. These therapies have been clinically neglected for a long time, though they have been found to have very negligible side effects. Considering this, we decided to check the epigenetic effect on cancer cells of one of the most promising natural compounds, vitamin C. We treated ECM-detached MCF-7 and HeLa cell lines with 5-aza-dc as a positive control and vitamin C for 6 Days. In the ECM-detached MCF-7 cell line, vitamin C treatment-induced NKG2D ligands and significantly downregulated *DNMT*s expression and the global DNA methylation (5mC) level. However, vitamin C treatment-induction of NKG2D ligands did not significantly affect *DNMT3b* mRNA levels in ECM-detached HeLa cell line.

Nevertheless, vitamin C treatment downregulated *DNMT1/3a* mRNA expression in the HeLa cell line. A previous study may explain this controversy, for it was demonstrated there that ascorbate (vitamin C) could inhibit only DNMT enzyme activity in melanoma cells [54]. Additional analyses showed that DNMTi treatment slightly reduced DNMT expression [55].

Nevertheless, 5mC level assessment revealed that both cell lines had reduced global methylation levels after vitamin C treatment. Additionally, the MSPCR result for the *MICB* gene showed that vitamin C reduced the methylation signal only in the MCF-7 *but* not in the HeLa cell line. However, in both cell lines, *MICA* gene methylation was not detected in MSPCR.

Hence, our overall results demonstrate that the two cell lines showed almost similar outcomes. MICA/B gene and surface protein expression were induced in DNMTi- and vitamin C-treated MCF-7 cell lines, with significant downregulation of DNMT and global methylation (5mC) levels. This means that MICA/MICB genes may have been suppressed or downregulated by epigenetic mechanisms in ECM-detached MCF-7 cell lines. On the other hand, the DNMTi- and vitamin C-treated ECM-detached HeLa cell line had reduced global DNA methylation. Though treatment reduced *DNMT1/3a* expression while not affecting *DNMT3b*, DNMTi and vitamin C induced MICA/B expression, suggesting that the *MICA/B* genes in the ECM-detached HeLa cell line are probably regulated by other epigenetic mechanisms. A previous study also suggested this probability, having shown that *MICA/B* promoters are silenced by histone hypo-acetylation [56]. Even DNMTi treatment can induce 5hmC [57], which is also methylated; hence, hydroxyl methylation in MSPCR bands may have been present in the vitamin C-treated ECM-detached HeLa cell line, such that the reduction of methylation was not shown after treatment.

## 4. Methods

### 4.1. Cell Culture and Viability Assay

Poly-HEMA gel was purchased from Sigma, dissolved in 95% ethanol at 65 °C, and incubated overnight. Cells were then seeded in a poly-HEMA- (lot no. SLBV7383, Sigma, India) coated plate. MCF-7 and HeLa cell lines were collected and grown and subcultured in DMEM media (UFS Biotech, Riyadh, Saudi Arabia) supplemented with 10% FBS and 1% penicillin and incubated in a CO_2_ incubator. Upon 85–90% confluence, cells were trypsinized and seeded in poly-HEMA-coated 96-well plates and incubated overnight to ensure that cells were healthy without any contamination. Then, cells were treated with 5-aza-dc (lot no. S1782, Selleckchem, Houston, TX, USA) at 500 nM, 1.5 µM, and 3 µM for 48 h. For vitamin C (lot no. S13114, Selleckchem) treatment, cells were treated at 5 µM, 10 µM, 15 µM, 20 µM, 25 µM, and 30 µM. After 48 h, WST-1 was added, and a cell viability assay was performed with a BioTek ELISA microplate reader at 450 nm. Based on the cell viability assay, we selected doses of 5-aza-dc of 60 µM and 240 µM for vitamin C treatment for 6 days.

### 4.2. Metabolites Extraction

Metabolites were extracted from attached and detached cells with methanol: acetonitrile: water at a ratio of 2:2:1 (*v/v*). Then, 1 mL of ice-cold solvent was added to the cell supernatant, followed by quick vortexing for 30s and incubation for 1 h at −20 °C. The supernatant was then spun for 15 min at 13,000 rpm at 4 °C, after which it was removed and the sample was dried, reconstituted in 100 μL of acetonitrile: water (1:1, *v*/*v*), vortexed for 10 min, and spun for 15 min at 13,000 rpm at 4 °C to remove insoluble debris. Finally, supernatants were taken for LC–MS/MS [58].

### 4.3. Analysis of HPLC Coupled LC–MS/MS

For LC–MS/MS, 10 µL of the sample was taken for injection into the HPLC column of an LTQ XL™ linear ion trap instrument (Thermo Fisher Scientific, Waltham, MA, USA). The full scan range was chosen from 100 to 1000 m/z, with spray voltage at −3.0 kV and capillary voltage at 4.0 V. The temperature was fixed at 270 °C. Helium was used as a buffer gas, nitrogen as a sheath gas. The flow rate was fixed at 40 arbitrary units. Next, the obtained data were processed with XCMS online server data processing software [18].

### 4.4. Quantitative Real-Time Reverse Transcription PCR

Total RNA was extracted using a PureLink™ RNA Mini Kit (lot no. 1944999, Thermo Fisher Scientific). Then, 100 ng/uL RNA was transcribed into cDNA using a high-capacity cDNA synthesis kit (lot no. 00656567, Applied Biosystems, Waltham, MA, USA). *AHCY*, *MICA*, *MICB*, and *DNMTs* primers sequences were obtained from the UCSC genome browser. One-carbon metabolic gene primers were obtained from other studies [47,48,49,59,60,61,62]. All the primer sequences are enlisted in Table 1. qRT-PCR was carried out by PowerUp SYBR Green Master Mix (lot no. 1805029, Applied Biosystems). The conditions for qRT-PCR were: 50 °C for 2 min, 95 °C for 2 min, 95 °C for 15 s, and 60 °C for 1 min. After that, qRT-PCR was performed in an ABI 7300 Prism. The numbers of transcripts were normalized with RPLP0 and calculated by applying the 2^−(ΔΔCt)^ method [63].

### 4.5. Quantitative Assay of DNA Methylcytosine (5mC) Level

DNA was extracted using an easy DNA kit (lot no. 155152507, MQ Genomic DNA isolation Kit, Molequle On, Auckland, New Zealand). The 5mC kit was purchased from Abcam (lot no. ab117128, Abcam, Trumpington, UK). The extracted DNA was seeded for binding the DNA to the assay well. Then, the wells were washed and the capture antibody was added. After that, the wells were rewashed, and detection antibody, enhancer solution, and developing solution were added. Finally, absorbance was observed at 450 nm. The whole procedure and data calculation were performed as per the manufacturer’s protocol.

### 4.6. Flow Cytometry

Both HeLa and MCF-7 cell lines were seeded in poly-HEMA-coated 24-well plates and treated with 5-aza-dc at 60 µM and 240 µM for vitamin C treatment for 6 days. Then, the collected cells were washed with 1X PBS, followed by thorough mixing with 100 uL 2% FBS. Anti-Hu MICA/B Ab (lot no. 2115636, Invitrogen, eBioscience^TM^, Waltham, MA, USA) was diluted as 1:100 in 2% FBS for staining. Stained samples were put in a shaker for 1 h, followed by 1X PBS wash. After that, the surface expression of MICA/B was analyzed by Guava easyCyte HT.

### 4.7. Bisulfite Conversion and Methylation-Specific PCR (MSP)

Using an EpiJET DNA bisulfite conversion kit (lot no. 00596381, Thermo Fischer Scientific, Waltham, MA, USA), bisulfite conversion of extracted DNA was performed according to the supplier’s protocol. The bisulfite-modified DNA template was used for further PCR reactions using the DNA Master Mix Kit (Molequle On). For PCR reactions, whole gene sequences were selected for both *MICA/B* genes and submitted to Metprimer online software (https://www.urogene.org/methprimer/, accessed on 15 January 2020) for methylated and unmethylated primer design (listed below, Table 2). During the primer design, CpG islands were selected. Bisulfite-converted DNA was taken for qPCR-mediated amplification using MSP primers; the qRT-PCR conditions were 50 °C for 2 min, 95 °C for 2 min, 95 °C for 15 s, and 60 °C for 1 min, and amplified products were visualized in the UVP iBOX 500 imaging system after gel electrophoresis (SCIE PLAS, Cambridge, UK). The signal intensity (IntDen) of the MSPCR amplicon was measured using Image J software.

### 4.8. Statistical Analysis

The statistical analysis was carried out by multiple *t*-tests or two ANOVA mixed models using GraphPad Prism 9 software. The Holme–Sidak and Tukey test methods were chosen, and the alpha was set as ≤0.5 for considering statistical significance. The data were presented as mean ± SEM.

## 5. Conclusions

ECM-detached cancer cells possess high levels of one-carbon metabolites that mediate the induction of global methylation in these conditions. Higher global DNA methylation levels may suppress one of the major NK cell immune ligands-MICB. Hence, targeting the one-carbon metabolic pathway and/or global methylation levels constitutes a beneficial strategy for chemotherapy outcomes in patients.

## Figures and Tables

**Table 1:** List of qRT-PCR primers.

*Gene*	Forward Primer (5′–3′)	Reverse Primer (5′–3′)
*MICA*	CCT GCA ATC CCA GCA CTT TG	ATT CAC CAC CAA GCC CGT CT
*MICB*	CAC GTT CGC CCT TTG TTC AG	GGA GGC AGA GGT TGC AGT GA
*DNMT1*	CAG CAA CGG GCA GAT GTT TC	CGG AGG GGTG CTTTGT AGA TG
*DNMT3a*	CTA CGC ACC ACC TCC ACC AG	CAA TGT TCC GGC ACT TCT GC
*DNMT3b*	GAG TCC ATT GCT GTT GGA ACC G	ATG TCC CTC TTG TCG CCA ACC T
*MAT2A*	ATGAACGGACAGCTCAACGG	CCAGCAAGAAGGATCATTCCAG
*MAT2A*	ATGAACGGACAGCTCAACGG	CCAGCAAGAAGGATCATTCCAG
*AHCY*	GCTGGAAGTTGGAGTTCTCGC	GTCCTCCCGCTGCTGTCA
*BHMT*	GTC ATG CAG ACC TTC ACC TTC TA	CTC CTT CAT GAG CTT CAC TG
*CBS*	ACA TGA CCA AGT TCC TGA GC	GCC ACG AAG TTC AGC AAG TC
*DHFR*	GTCCTCCCGCTGCTGTCA	GCCGATGCCCATGTTCTG
*MTHFD1*	CGTGGGCAGCGGACTAA	CCTTATTTGCGCGGAGATCT
*MTHFR*	GGCCATCTGCACAAAGCTAAG	AACTCACTTCGGATGTGCTTCAC
*TYMS*	TCTGGAAGGGTGTTTTGGAG	CCTCCACTGGAAGCCATAAA
*SHMT1*	AGGAAAGGAGTGAAAAGTGTGGAT	GACACCAGTGTCGCTCTGGATCTG

**Table 2:** List of MSPCR primers.

*Gene*	Forward Primer (5′–3′)	Reverse Primer (5′–3′)
*MICA* methylated	TTA TTG TTA GTA ACG TTG TGC GC	AAC CTA AAA CAA AAA CCA ACT TCG
*MICA* unmethylated	TTA TTG TTA GTA ATG TTG TGT GTG G	ACC TAA AAC AAA AAC CAA CTT CAA A
*MICB* methylated	GTT GGG ATT ATA GAG GTG AGT TAT C	CTC AAA AAA ACT AAT TTA TCC GAA
*MICB* unmethylated	TGT TGG GAT TAT AGA GGT GAG TTA TT	CCT CAA AAA AAC TAA TTT ATC CAA A

## Data Availability

The data presented in this study are available on request from the corresponding author.

## Supplementary Materials

The following are available online at https://www.mdpi.com/article/10.3390/metabo12030267/s1, Figure S1: Cell viability assay for 5-aza-dc-treated monolayer and spheroid cell lines; Figure S2: 5-aza-dc-mediated expression of NKG2DLs and status of DNA methylation in HeLa cell lines; Figure S3: 5-aza-dc-mediated expression of NKG2DLs and status of DNA methylation in MCF-7 cell lines.
