# Supplementary material for: Untargeted Metabolomics Showed Accumulation of One-Carbon Metabolites to Facilitate DNA Methylation during Extracellular Matrix Detachment of Cancer Cells
Source: Metabolites. 2022 Mar 21;12(3):267. doi: 10.3390/metabo12030267 (PMC8951017; doi:10.3390/metabo12030267)
Supplement: Supplementary file 1 [file metabolites-12-00267-s001.zip › metabolites-1629350-supplementary.pdf]

Supplemetary Figure S1

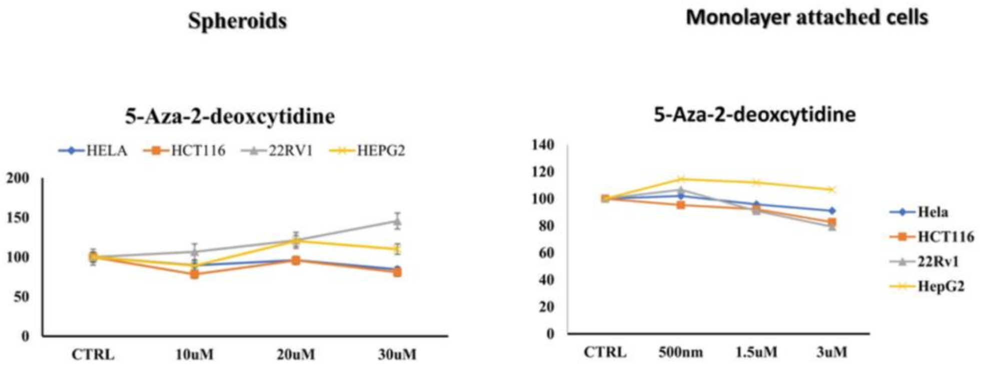

Supplementary Figure S1 1*10^5 cells were plated on normal cell culture plate (A and B) and cells plated in ultra low attachment plate (ul) allowed to grow for one week and treated with inhibitor for 48hr their percentage of survival was measured using WST1 assay

Supplementary Figure S2

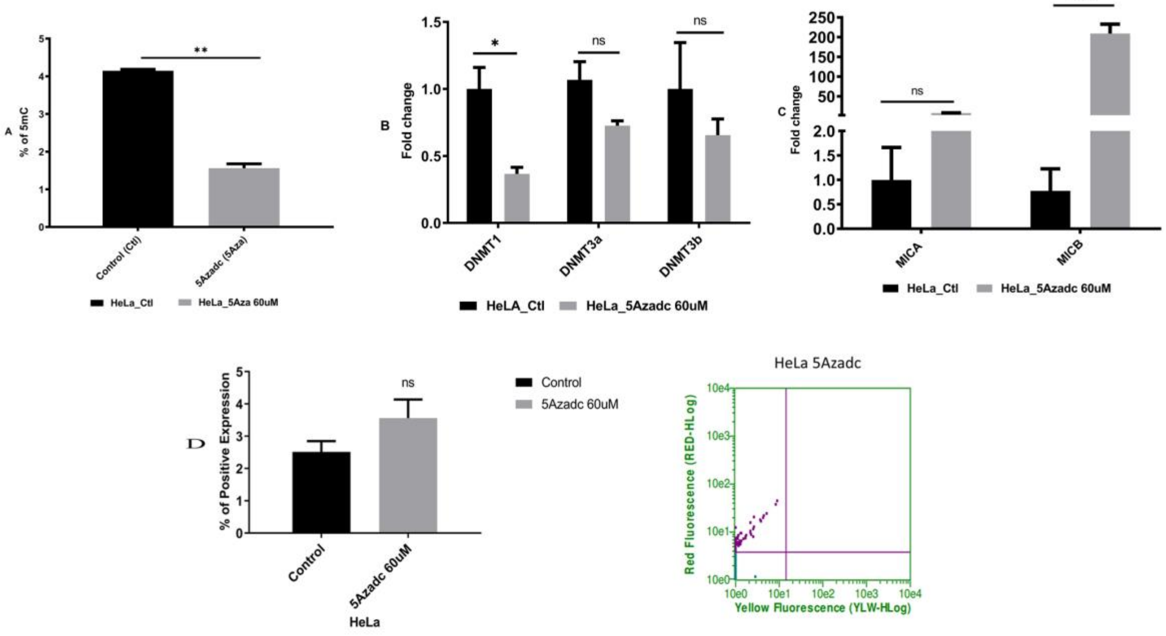

Supplementary Figure S2: **5Azadc mediated NKG2DLs expression and status of DNA methylation in HeLa cell lines.** A. Reduced percentage of 5mC level after 5Azadc treatment in 6days detached cells. B. 5azadc treatment reduced the DNMTs mRNA expression in 6 days ECM detached cell lines. C. MICA/B mRNA expression upregulation after 5Azadc treatment in 6 days ECM detached HeLa cells. D. HeLa showed induced expression of MICA/B ligand expression after 5Azadc in 6 days ECM detached cell compared to untreated ECM detached cell. The data were presented as Mean±SEM; ns, not significant; *P<0.05; **P<0.01; ***P<0.001 and ****P<0.0001.

Supplementary Figure S3

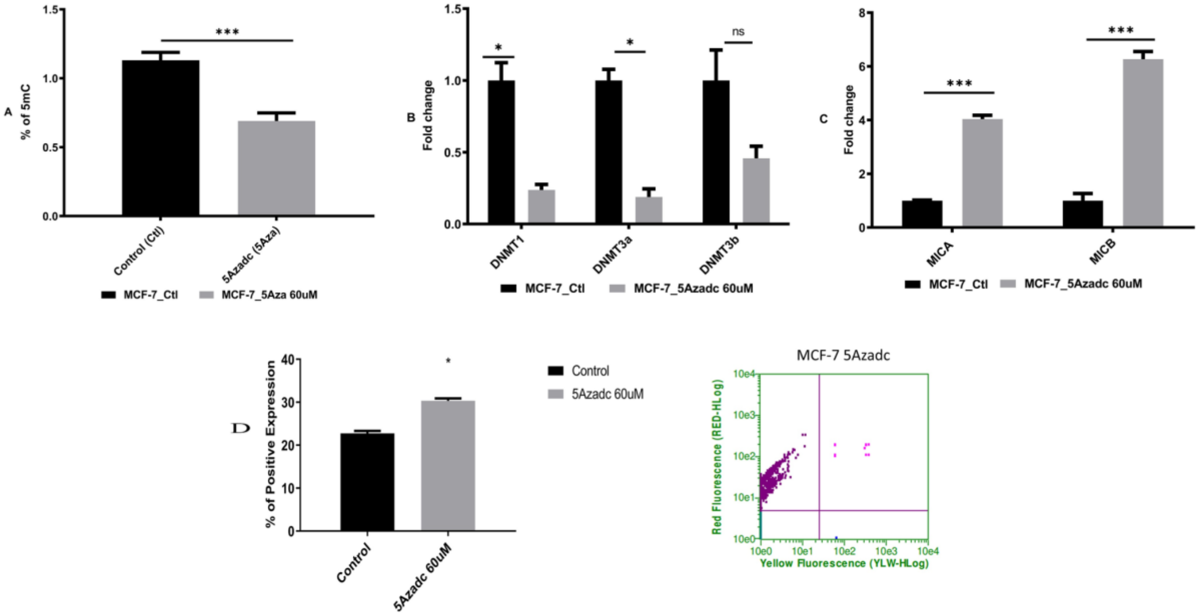

Supplementary Figure S3: **5Azadc mediated NKG2DLs expression and status of DNA methylation in MCF-7 cell lines.** A. The percentage of 5mC activity reduced after 5Azadc treatment in 6 days detached MCF-7 cells. B. Fold change of DNMTs mRNA expression. C. Fold change of MICA/B mRNA expression after 5Azadc treatment in 6 days MCF-7 cells. D. MCF-7 showed induction of surface MICA/B ligand expression after 5Azadc in 6 days ECM detached cell compared to untreated ECM detached cell. The data were presented as Mean±SEM; ns, not significant; *P<0.05; **P<0.01; ***P<0.001 and ****P<0.0001.
